# *Clostridium difficile* infection in an Iranian hospital

**DOI:** 10.1186/1756-0500-5-159

**Published:** 2012-03-21

**Authors:** Mohammad Jalali, Farzin Khorvash, Keith Warriner, J Scott Weese

**Affiliations:** 1 School of Nutrition and Food Sciences and Food Security Research Center, Isfahan University of Medical Sciences, Isfahan, Iran; 2 Department of Infectious Disease, Al-Zahra Hospital, Isfahan University of Medical Sciences, Isfahan, Iran; 3 Department of Food Sciences, University of Guelph, Guelph, ON, Canada; 4 Department of Pathobiology, University of Guelph, Guelph, ON, N1G 2W1, Canada

**Keywords:** *Clostridium difficile*, Diarrhea, Nosocomial, Infectious disease, Gastroenterology

## Abstract

**Background:**

*Clostridium difficile* infection (CDI) is an important cause of morbidity and mortality internationally, yet there are important regional differences in the epidemiology and microbiology of disease. Most reports have come from North America and Europe, with limited information from other regions, including the Middle East. Given the changes in the epidemiology of CDI in developed countries, particularly associated with the dissemination of hypervirulent epidemic clones, an understanding of the epidemiology and microbiology of CDI in diverse regions is warranted. This study involved collection of stool samples from individuals with diarrhea at the Isfahan University of Medical Sciences Teaching Hospital, Isfahan, Iran, between October 2010 and March 2011. Selective enrichment culture for *C. difficile* was performed and isolates were characterised using ribotyping, PCR for the detection of *tcdA, tcdB* and *cdtB* genes, and *tcdC* sequence analysis.

**Findings:**

*Clostridium difficile* was isolated from 19/89 (21%) stool samples of 17/86 (20%) patients. 13/17 (77%) cases of CDI were hospital-associated. Patients with CDI were significantly older (43 ± 28y) than those with non-CDI diarrhea (24, ± 26y)(*P* = 0.018). All isolates were toxigenic, and possessed genes encoding for toxins A and B. Six (32%) of 19 isolates also possessed *cdtB*. Twelve ribotypes were identified. Ribotype 078/toxinotype V was most common, accounting for 4 (21%) of isolates. A single isolate of a different toxinotype V ribotype was identified, as was a toxinotype XXIV isolate. The remaining isolates consisted of 9 different toxinotype 0 ribotypes.

**Conclusions:**

CDI is an important cause of diarrhea in patients in this hospital. The diversity of ribotypes was striking, and the number of different types suggests the presence of a broad range of strains in the community, the hospital or both. The predominance of toxinotype V strains, which have been associated with community-associated disease and food animals, was unexpected and possible sources of this type require further investigation.

## Findings

*Clostridium difficile* is a leading cause of hospital-associated and antimicrobial-associated diarhea, and is of significant concern because of the increasing morbidity, mortality and relapse rates [1], along with the emergence of community-associated disease [2]. Some of these clinical and epidemiological changes have been associated with dissemination of hypervirulent clones, particularly ribotype 027 (toxinotype III, North American pulsotype (NAP)1)[3] and to a lesser degree ribotype 078 (toxinotype V, NAP7/8) [4,5]. *Clostridium difficile* infection (CDI) has been reported throughout much of the world, but most data come from developed countries in North America and Europe. Limited information is available regarding the role of *C. difficile* in diarrheic hospital patients in people in Iran or other Middle Eastern countries, or about *C. difficile* strains that are involved. Therefore, the aim of present study was to determine the prevalence of *C. difficile* in diarrheic patients in hospital in Isfahan, Iran, and to characterize isolates.

The study was conducted at the Isfahan University of Medical Sciences Teaching Hospital, Isfahan, Iran, the largest tertiary care medical centre in the region with 800 beds, three intensive care units and all major clinical specialties. Aliquots of stool specimens that had been collected from diarrheic patients between October 2010 and March 2011 for other diagnostic testing were studied. Both patients that were admitted to hospital with diarrhea and those who developed diarrhea during hospitalization were included. Diarrhea was defined as watery, loose or unformed stool passed at a frequency of three times or more per 24 hours. Demographic and basic medical information were collected from the medical record. Cases were classified as community associated (CA) if the onset of symptoms occurred prior to, or within 48 h of admission and if they had not been hospitalized in the preceding 3 months [6]. Cases were classified has hospital-associated (HA) if the onset was greater than 48 h after admission. Cases that had diarrhea at admission or developed diarrhea within the first 48 h of admission but had been hospitalized within the preceding 4 weeks were classified as hospital-associated, community-onset (HA-CO). Studies such as this are exempt from ethics board review at Isfahan University and instead require approval of the Isfahan University of Medical Sciences Vice Chancellor for Research, which was obtained.

Selective enrichment culture was performed. Approximately 5 g of stool was inoculated into 25 ml of *C. difficile* selective enrichment broth containing 40 g/l proteose peptone, 5.0 g/l disodium hydrogen phosphate, 0.1 g/l magnesium sulphate, 2.0 g/l sodium chloride, 6.0 g/l fructose and 1.0 g/l sodium taurocholate supplemented with cysteine hydrochloride, norfloxacin and moxalactam, and anaerobically incubated at 37 C for 5–7 days. Two ml of each culture were then added to an equal volume of absolute ethanol, mixed and left at room temperature for 1 h. Alcohol shocked cultures were then centrifuged (4000 rpm/10 min) and the pellet was streaked onto *C. difficile* moxalactam norfloxacin (CDMN) agar (Oxoid) and incubated anaerobically at 37 C for 24-48 h. Suspect colonies were subcultured and identified as *C. difficile* on the basis of characteristic colony morphology, odour, Gram stain morphology and L-proline aminopeptidase test (Prodisk, Remeb, Lenexa, KS, USA). All isolates were screened for the presence of genes encoding toxin A (*tcdA*), toxinB *(tcdB*), the binding component of CDT (*cdtB*) and triose phosphate isomerase (*tpi*) as have been previously described [7-9]. Isolates were also subjected to PCR ribotyping [10], toxinotyping[11] and *tcdC* sequence analysis [12]. For ribotyping, ribotypes were assessed visually and compared to an internal collection of ribotypes from over 3000 isolates from humans and animals. When a ribotype pattern was known to be a recognized international ribotype through previous typing of reference strains from the HPA Anaerobic Reference Laboratory (http://www.webcitation.org/64HSI8B56) (Cardiff, UK), the appropriate numerical designation (i.e. 078) was used. Otherwise, internal nomenclature was used.

Categorical comparisons were performed using Fisher’s exact test while t-test was used for continuous data. A *P* value of <0.05 was considered significant.

Eighty-six diarrheic patients were enrolled. *Clostridium difficile* was isolated from 19/89 (21%) of stool samples from 17/86 (20%) patients; 8/36 (22%) women and 9/50 (18%) men (*P* = 0.78). 13/17 (76%) cases were HA-CDI while the other 4 (24%) were CA-CDI. Patients with CDI were significantly older (43 ± 28y) than those with non-CDI diarrhea (24, ± 26y)(*P* = 0.018). Five (29%) CDI patients were over the age of 65 compared to 6/69 (8.7%) without non-CDI diarrhea (*P* = 0.037). Fifteen (88%) of CDI patients had received antimicrobials prior to the onset of diarrhea, compared to 58 (84%) others (*P* = 1.0).

Two patients had separate episodes of diarrhea 62 and 33 days apart, and the same ribotype was isolated from each episode. Follow-up data were limited but this represents a minimal recurrence rate of 12%. Two (12%) patients with CDI died during hospitalization, however death was only attributed to CDI in 1 (5.9%) individual.

**Figure 1:**
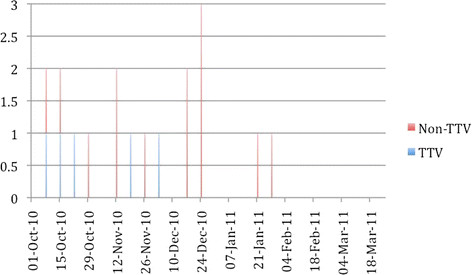
Temporal distribution of *Clostridium difficile* infection caused by toxinotype V (TT V) and non toxinotype V (Non TT V) isolates

All isolates possessed genes encoding for toxins A and B, and 12 different ribotypes were identified (Table 1). In addition to *tcdA* and *tcdB*, six (32%) isolates also possessed *cdtB*. Four of these were ribotype 078 and toxinotype V, and contained a 39 bp deletion and C184T nonsense mutation in *tcdC*. One ribotype was a toxinotype V isolate that differed from ribotype 078 but had the same *tcdC* deletion and mutation. Patients carrying these five isolates were admitted over a period of approximately 2 months, with no more than one per week (Figure 1). The other *cdtB* positive isolate was a toxinotype XXIV strain that contained an 18-bp *tcdC* deletion in *tcdC* but not the upstream truncating mutation that is characteristic of the toxinotype III hypervirulent ribotype 027/NAP1. The remaining 13 isolates from 11 patients were toxinotype 0 strains consisting of 9 different ribotypes, including ribotype 014 and 8 ribotypes not previously encountered in this laboratory.

**Table 1:** Ribotype and toxinotype data for *Clostridium difficile* isolated from diarrheic individuals in an Iranian hospital

Ribotype	n	Toxin gene profile	Toxinotype
078	4	*tcdA, tcdB, cdtB*	V
IR1	1	*tcdA, tcdB, cdtB*	V
014	1	*tcdA, tcdB*	0
IR2	1	*tcdA, tcdB, cdtB*	XXIV
IR3	1	*tcdA, tcdB*	0
IR4	3	*tcdA, tcdB*	0
IR5	1	*tcdA, tcdB*	0
IR6	1	*tcdA, tcdB*	0
IR7	1	*tcdA, tcdB*	0
IR8	1	*tcdA, tcdB*	0
IR9	1	*tcdA, tcdB*	0
IR10	1	*tcdA, tcdB*	0

All 4 patients from which ribotype 078 was isolated were from rural areas, but had hospital-associated diarrhea. The mean age of patients with ribotype 078 was 29.2y compared to 49.1y for other ribotypes, but this was not statistically significant (*P* = 0.25)

The prevalence of CDI (21%) was consistent with many studies of hospitalized individuals in other regions, albeit higher than two recent Iranian studies that reported isolation of toxigenic *C. difficile* from 6.1-6.8% of stool samples [13,14]. Other regional studies have reported 4.6-9.5% prevalence in two studies from Saudi Arabia [15], 10.5% in Kuwait [16], and 13.7% from Jordan [17]. However, limited emphasis should be placed on comparisons of prevalence because of differences in study methodology. The key finding is that CDI appears to be common in patients in Iranian hospitals, and may be underdiagnosed since testing appears to be rarely performed, in part because of limited access to commercial assays.

As is consistent with other reports, CDI was associated with older individuals. However, in this study, the CDI patients were younger (mean 43y) compared to most other reports [1,18] and less than 30% of CDI patients were over the age of 65. Reasons for this difference are not apparent. Classically, CDI has been considered a disease predominantly of elderly individuals, however there are increasing reports of CDI in younger individual, including people that would be considered at low risk of infection [19-21]. Only limited investigation of risk factors was performed here based on the available data. There was no association between antimicrobial use and CDI in these patients, however antimicrobial use was very common in both CDI and non-CDI groups, and the role of antimicrobials in CDI can certainly not be dismissed by these data.

Community-associated CDI is being more commonly reported in some regions, often associated with milder disease in younger individuals and people with little or no antimicrobial exposure [2,20,22]. In this study, 23% of cases were classified as CA-CDI, and all involved different toxinotype 0 strains. While the majority of cases were therefore associated with hospitalization, it cannot be stated with certainty that exposure occurred in hospital since testing of patients at admission, prior to the onset of disease, would be required to ensure that they were not carrying *C. difficile* at the time of admission. Since there was a wide diversity in types and little evidence of temporal clustering (Figure 1), a point-source of hospital exposure was not apparent. Hospital-associated CDI could have occurred from exposure of different patients to different strains in hospital, but also could have been the result of development of disease from *C. difficile* that was resident in the intestinal tract at the time of admission.

The wide diversity of ribotypes was interesting. Toxinotype 0 strains predominated, yet only 1 isolate (ribotype 014) was consistent with major reported human types. The diversity of ribotypes within toxinotype 0 strains and the differences between common toxinotype 0 ribotypes in this versus other studies was interesting. The relatively high prevalence of ribotype 078 was consistent with two studies from Kuwait that identified ribotype 078 as one of the predominant types [23,24]. Additionally, the one other toxinotype V isolate is likely closely related to ribotype 078, and the overall prevalence of toxinotype V strains was 29%. Recent studies have reported increases in the prevalence of ribotype 078 or toxinotype V strains in humans, particularly in community-associated disease [19,25]. This strain can also be found commonly in food animals and food [26-28], leading to concern that it might be foodborne or zoonotic [29]. While all patients with ribotype 078 were from rural regions, disease onset was in hospital and the origin of infection cannot be discerned. Further, no information about animal or food contact is available, and no data regarding *C. difficile* in animals or food are available for this region. Therefore no assumptions can be made regarding potential sources of this strain. The four patients with ribotype 078 were admitted over a 37 day period (Figure 1), and the potential that these represent a small hospital-associated cluster cannot be dismissed. Ribotype 027, a toxinotype III strain associated with hypervirulent disease and epidemics internationally [30], was not identified in any patient. The diversity in ribotypes is consistent with the only available Iranian study, which reported 28 different ribotypes amongst 178 isolates [14]. That study did not compare strains to internationally recognized reference strains and did not perform other typing methods, so detailed comparison with these data is impossible.

This study indicates that *C. difficile* might be an important enteric pathogen in patients in Iranian hospitals. Efforts must be undertaken to diagnose CDI to allow for targeted therapy and to provide a better understanding of this important disease. While some aspects identified here are consistent with reports of CDI in other areas, further study of the epidemiology and microbiology of CDI in this region is required to explore some apparent differences, such as the younger age distribution and the predominance of ribotype 078. While ribotype 027 was not identified, further surveillance is required to determine whether this strain is indeed present in the country and to monitor for its emergence.

## Competing interests

The authors declare that they have no competing interests.

## Authors’ contributions

MJ, JSW and KW designed the study. FK coordinated sample and medical record data collection. MJ performed the laboratory tests under the direction of JSW. All authors contributed to writing and reviewing the final manuscript. All authors read and approved the final manuscript.
